# p18 inhibits reprogramming through inactivation of Cdk4/6

**DOI:** 10.1038/srep31085

**Published:** 2016-08-03

**Authors:** Shaohua Zhu, Jiani Cao, Hongyan Sun, Kun Liu, Yaqiong Li, Tongbiao Zhao

**Affiliations:** 1 School of Biological Sciences, University of Science and Technology of China, Hefei 230026, China; 2 State Key Laboratory of Stem Cell and Reproductive Biology, Institute of Zoology, Chinese Academy of Sciences, Beijing 100101, China; 3 Graduate University of Chinese Academy of Sciences, Beijing 100049, China

## Abstract

Pluripotent stem cells (PSCs), including embryonic and induced pluripotent stem cells (iPSCs), show atypical cell cycle regulation characterized by a high proliferation rate and a shorter G1 phase compared with somatic cells. The mechanisms by which somatic cells remodel their cell cycle to achieve the high proliferation rate of PSCs during reprogramming are unclear. Here we identify that the Ink4 protein p18, which is expressed at high levels in somatic cells but at low levels in PSCs, is a roadblock to successful reprogramming. Mild inhibition of p18 expression enhances reprogramming efficiency, while ectopic expression of p18 completely blocks reprogramming. Mechanistic studies show that expression of wild-type p18, but not a p18^D68N^ mutant which cannot inhibit Cdk4/6, down-regulates expression of Cdk4/6 target genes involved in DNA synthesis (TK, TS, DHFR, PCNA) and cell cycle regulation (CDK1 and CCNA2) and thus inhibits reprogramming. These results indicate that p18 blocks reprogramming by targeting Cdk4/6-mediated cell cycle regulation. Taken together, our results define a novel pathway that inhibits somatic cell reprogramming, and provide a new target to enhance reprogramming efficiency.

Somatic cells can be reprogrammed into induced pluripotent stem cells (iPSCs) by ectopic expression of certain combinations of transcription activation factors, such as Oct4, Sox2, Klf4, and c-Myc, or Oct4, Sox2, Nanog, and Lin28^1^,^2^,^3^,^4^,^5^. iPSCs are molecularly and functionally similar to embryonic stem cells (ESCs), in terms of pluripotent gene expression and the ability to support differentiation into three embryonic germ layers^1^,^2^,^3^,^4^,^5^. Furthermore, iPSCs can be generated using patient somatic cells directly without destroying human embryos; thus iPSCs hold great promise for regenerative medicine while circumventing ethical problems and immune rejection^6^. However, the efficiency of iPSC generation is still very low, indicating the existence of reprogramming barriers.

PSCs show atypical cell cycle regulation, characterized by a higher proliferation rate and a shorter G1 phase compared with somatic cells^7^,^8^. It has been reported that cell cycle regulatory pathways and the pluripotency network are closely linked to maintain ESC identity^9^,^10^,^11^,^12^. During the reprogramming process, somatic cells undergo a series of changes in cell cycle regulation, including relaxation of the G1 checkpoint and acceleration of the cell cycle. Facilitating the G1/S and G2/M transitions and speeding up the cell cycle are assumed to enhance the ability to generate iPSCs. As the cell cycle is mainly restricted at the G1 checkpoint in somatic cells, key cell cycle regulators that function during the G1/S transition are proposed to play crucial roles in reprogramming.

In this study, we aimed to identify reprogramming barriers from the perspective of cell cycle regulation. We screened a number of cell cycle regulators that are differentially expressed in somatic cells and PSCs and identified p18 as a critical factor that restricts reprogramming. We demonstrated that p18 suppresses iPSC induction by inhibiting Cdk4/6 activity. Mild inhibition of p18 expression enhances reprogramming efficiency. In contrast, ectopic expression of p18 leads to decreased expression of Cdk4/6 target genes involved in DNA synthesis and cell cycle regulation, and thus blocks reprogramming.

## Results

### The expression of p18 is decreased in pluripotent stem cells

**Figure 1:**
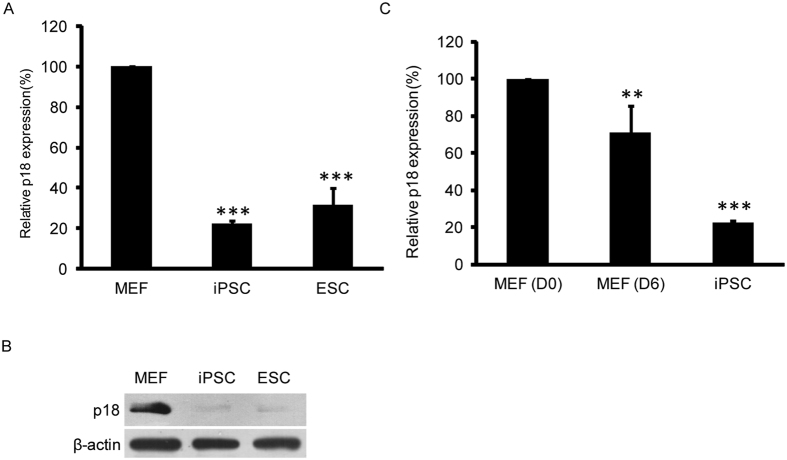
p18 expression is inversely correlated with pluripotency. (**A)** Relative expression of p18 mRNA in MEF, ESC, and iPSC. The results are presented as the mean ± SD of three independent experiments; ***P < 0.001. (**B**) Expression of p18 protein in MEF, iPSC, and ESC. Actin serves as a loading control. (**C)** Relative expression of p18 mRNA in MEF, reprogramming cells at day 6, and estabolished iPSC. The results are presented as the mean ± SD of three independent experiments; **P < 0.01; ***P < 0.001.

To screen for crucial cell cycle regulators that restrict reprogramming, mRNA expression data of different ESC lines, iPSC lines and fibroblast lines were downloaded from the NCBI website (Supplementary Table 2). Then 37 genes involved in cell cycle checkpoint and cell cycle arrest regulations were analyzed for their differential expressions between pluripotent stem cells and somatic cells (Fig. S1). A number of genes including p18, a member of the Ink4 family, were differentially expressed in somatic cells and PSCs (Fig. S1). The Cdkn1a, Cdkn2a and Cdkn2b, which have been suggested as reprogramming barriers, were identified to be highly expressed in somatic fibroblasts (Fig. S1)^13^. qPCR and Western blot assays confirmed the limited expression of p18 in PSCs at both the mRNA and protein levels compared to mouse embryonic fibroblasts (MEFs) (Fig. 1A,B). We further monitored the expression of p18 during the reprogramming process and found a gradual decrease in p18 expression as reprogramming progressed (Fig. 1C). The inverse correlation between p18 expression and pluripotency suggests that p18 might be involved in regulation of somatic cell reprogramming.

The iPSC colonies were picked at reprogramming day 12 and maintained in ESC culture medium as previously described^14^ (Supplementary Fig. 2A). The established iPSCs were alkaline phosphatase-positive and morphologically indistinguishable from ESCs (Supplementary Fig. 2B). They were positive for the cell surface marker SSEA1, and expressed the pluripotency genes Pou5f1, Sox2, Nanog, Errb, Tcl, and Tbx at the same level as ESCs (Supplementary Fig. 2C,D). Furthermore, the iPSCs had normal karyotypes, and were able to differentiate into teratomas and contribute to chimeric mice, validating their pluripotency (Supplementary Fig. 2E–G).

### Mild inhibition of p18 decreases the G1:G2/M ratio and enhances reprogramming efficiency

**Figure 2:**
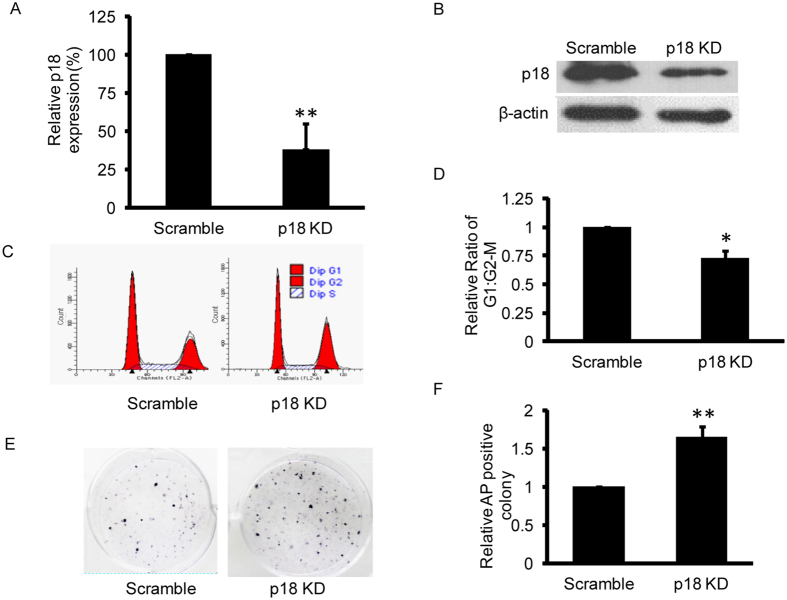
Inhibition of p18 expression facilitates reprogramming. (**A**) Transfection of p18-specific shRNA significantly inhibits p18 mRNA expression. Data are normalized to cells transfected with scramble shRNA and are shown as mean ± standard deviation (SD), n = 3, **P < 0.01. (**B)** Western blot analysis of whole cell extracts from MEFs transfected with scramble or p18-specific shRNA. Actin serves as a loading control. (**C**) Histograms of DNA contents in cells transfected with an expression vector containing either scramble or p18-specific shRNA. (**D**) p18 knockdown significantly decreases the proportion of cells in G1 phase. Data are normalized to cells transfected with scramble shRNA and are shown as mean ± SD; n=3, *P < 0.05. (**E**) Alkaline phosphatase (AP) staining of iPSC colonies. (**F**) p18 knockdown enhances reprogramming efficiency. AP-positive colonies were counted and normalized to cells transfected with scramble shRNA. Data are shown as mean ± SD; n = 3, **P < 0.01.

To test the mechanism by which p18 regulates reprogramming, we firstly investigated the effects of p18 down-regulation on reprogramming. We infected MEFs with lentivirus vectors carrying either scramble shRNA or specific shRNAs against p18. qPCR and Western blot analysis showed that the p18 expression level in MEFs was decreased after transfection with p18 shRNAs (Fig. 2A,B; Supplementary Fig. 3A). Accordingly, p18 knockdown significantly decreased the G1 cell population while increasing the G2/M cell population (Fig. 2C,D; Supplementary Fig. 3B). Next, to detect the effects of p18 on reprogramming, we infected MEFs with retrovirus vectors carrying Oct4/Sox2/Klf4/c-Myc (OSKM) coding sequences and lentivirus vectors carrying either scramble shRNA or specific shRNAs targeting p18. The resulting iPSC colonies were stained with alkaline phosphatase (AP) and quantified for reprogramming efficiency. The results showed that knockdown of p18 expression significantly enhanced the number of AP-positive colonies, indicating that the reprogramming efficiency is increased upon p18 inhibition (Fig. 2E,F).

### Ectopic expression of p18 increases the G1:G2/M ratio and blocks reprogramming

**Figure 3:**
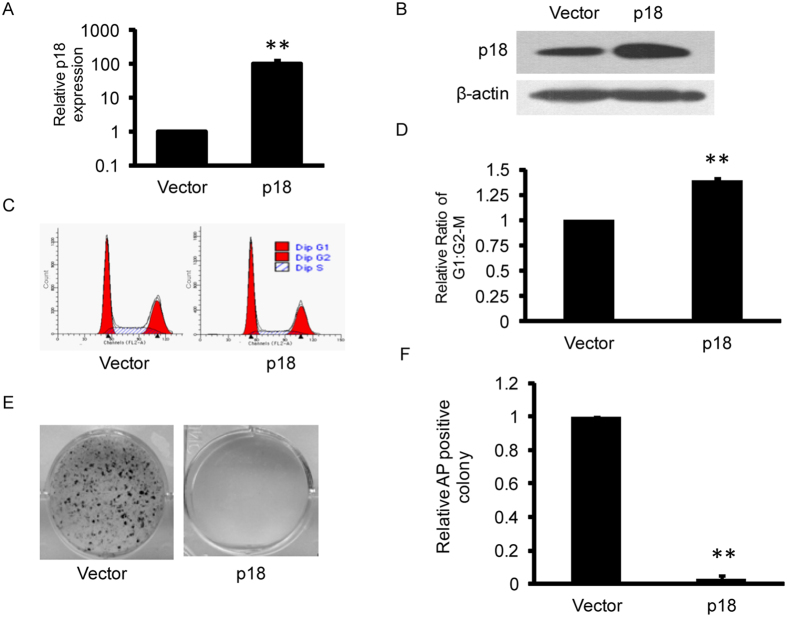
Overexpression of p18 blocks reprogramming. (**A**) Relative expression of p18 mRNA in MEFs transfected with empty vector or vector expressing p18 cDNA. The results are presented as the mean ± SD of three independent experiments; **P < 0.01. (**B**) Western blot analysis of whole cell extracts from MEFs transfected as in (**A**). Actin serves as a loading control. (**C**) Histograms of DNA contents in cells transfected as in (**A**). (**D**) p18 overexpression significantly increases the proportion of cells in G1 phase. Data are shown as mean ± SD; n = 3, *P < 0.05. (**E**) Alkaline phosphatase (AP) staining of iPSC colonies generated from control cells or cells overexpressing p18. (**F**) p18 overexpression blocks reprogramming. AP-positive colonies were counted and normalized to cells transfected with empty vector. Data are shown as mean ± SD; n = 3, **P < 0.01.

We next investigated the effect of p18 overexpression on reprogramming. We infected MEFs with retrovirus vectors that were either empty or carried the p18 coding sequence. qPCR and Western blot analysis showed that the p18 expression level was significantly elevated in MEFs infected with p18 expression vectors (Fig. 3A,B). Accordingly, p18 overexpression significantly increased the G1 cell population while decreasing the G2/M cell population (Fig. 3C,D). Next we infected MEFs with retrovirus vectors carrying OSKM and p18 coding sequences to detect the effect of p18 overexpression on reprogramming. In contrast to p18 knockdown, p18 overexpression completely blocked iPSC colony formation, indicating that p18 inhibits reprogramming (Fig. 3E,F).

### p18 blocks reprogramming by inhibiting Cdk4/6

**Figure 4:**
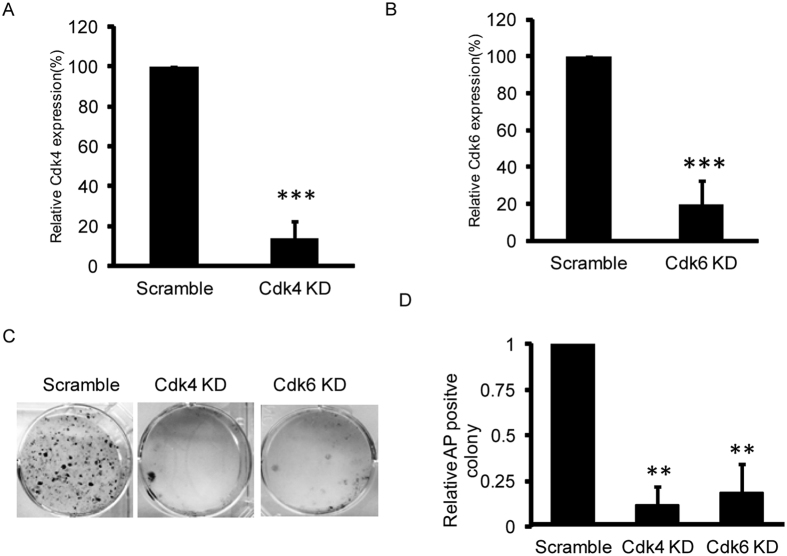
Knockdown of Cdk4 and Cdk6 blocks reprogramming. (**A)** Transfection with a Cdk4-specific shRNA significantly inhibits Cdk4 mRNA expression. Data are normalized to cells transfected with scramble shRNA and shown as mean ± SD; n = 3, ***P < 0.001. (**B**) Transfection with a Cdk6-specific shRNA significantly inhibits Cdk6 mRNA expression. Data are normalized to cells transfected with scramble shRNA and shown as mean ± SD; n = 3, ***P < 0.001. (**C**) AP staining of iPSC colonies generated from control cells (scramble), Cdk4 knockdown cells or Cdk6 knockdown cells. (**D**) Statistical analysis of the effects of Cdk4/6 inhibition on iPSC colony formation. Data are normalized to control cells and shown as mean ± SD; n = 3, **P < 0.01.

In somatic cells, p18 interacts with both Cdk4 and Cdk6 to prevent their interactions with D-type cyclins, thereby inhibiting their kinase activities and negatively regulating cell division^15^. To dissect the molecular mechanisms by which p18 restricts reprogramming, we studied the effects of the p18 target genes Cdk4 and Cdk6 on reprogramming. Knockdown of Cdk4 and Cdk6 expression significantly decreased reprogramming efficiency (Fig. 4A–D). This is in accordance with the inhibitory effects of p18, and indicates the possible involvement of Cdk4/6 in reprogramming.

**Figure 5:**
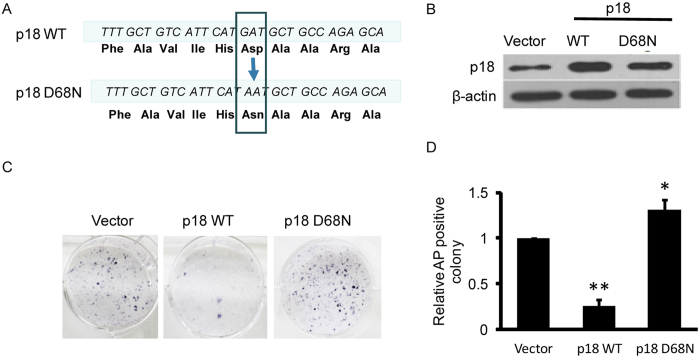
p18 inhibits reprogramming through Cdk4/6. (**A**) Sequences of wild-type (WT) and mutant (D68N) p18 proteins. (**B**) Western blot analysis of whole cell extracts from MEFs transfected with vector, WT p18 or D68N mutant p18. Actin serves as a loading control. (**C**) AP staining of iPSC colonies generated by overexpression of WT or D68N mutant p18. Transfection with empty vector serves as a control. (**D**) Statistical analysis of the effect of WT p18 or p18^D68N^ mutant expression on iPSC colony formation. Data are normalized to control cells (vector) and shown as mean ± SD; n = 3, *P < 0.05, **P < 0.01.

Mutation of Asp76 in human p18, which is equivalent to Asp68 in mouse p18, abolishes the ability of p18 to inhibit Cdk4/6^16^. To further investigate whether p18 inhibits reprogramming by targeting the Cdk4/6 pathway, we constructed retrovirus vectors carrying either wild-type mouse p18 or a D68N mutant (Fig. 5A,B). Interestingly, while overexpression of wild-type p18 significantly inhibited Cdk4/6 target gene expression, ectopic expression of the p18^D68N^ mutant increased the level of Cdk4/6 target gene expression above that in control cells (Supplementary Fig. 4A,B). This is possibly due to an effect of the interaction between p18^D68N^ and Cdk4^16^. We next infected reprogramming MEFs with empty vector and vectors carrying either wild-type or D68N mutant p18. As expected, expression of wild-type p18 significantly inhibited the reprogramming efficiency, while expression of p18^D68N^ mutant increased reprogramming efficiency dramatically (Fig. 5C,D). Together, these data indicate that p18 inhibits somatic cell reprogramming by targeting the Cdk4/6 pathway.

## Discussion

During the reprogramming process, somatic cells need to shorten the G1 phase and accelerate the cell cycle to match the high proliferation rate of PSCs. It was previously unclear how somatic cells regulated their cell cycles during reprogramming to facilitate the acquisition of pluripotency. In this study, we showed that p18 restricts somatic cell reprogramming by targeting the Cdk4 and Cdk6 pathways.

**Figure 6:**
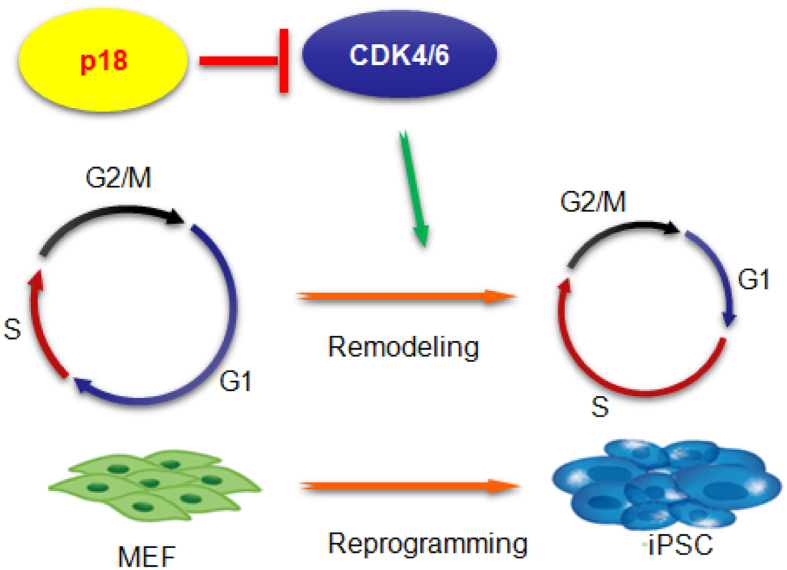
Schematic diagram showing how p18 inhibits reprogramming. The high expression of p18 in somatic cells inhibits Cdk4/6 activity, leading to compromised G1/S and G2/M transition. Somatic cell reprogramming is therefore blocked.

It has been proposed that G1/S and G2/M phase transitions can influence the fate of stem cells and are important for stem cell differentiation. In support of this hypothesis, recent studies showed that shortening the G1 phase in neural stem cells by increasing Cdk4/cyclinD1 delays neurogenesis and promotes the expansion of basal progenitors^17^. These data indicate that a shortened G1 phase might be involved in protecting stem cells from external signals that induce differentiation. Reprogramming of somatic cells to iPSCs is regarded as the reverse of differentiation, a biological process which also involves dramatic changes in the cell cycle^18^. Silencing of p53 significantly enhances reprogramming efficiency, suggesting that the p53 pathway serves as a barrier to reprogramming^19^. Further mechanistic analysis revealed that p53 inhibits reprogramming by inducing apoptosis, senescence or cell cycle arrest^5^,^20^. In contrast, we showed here that p18 blocks reprogramming through inhibition of the cell cycle regulators Cdk4 and Cdk6. p18 is a cyclin-dependent kinase (Cdk) inhibitor which has been identified as a negative regulator of the G1/S transition, and is extensively expressed in various kinds of tissue^21^,^22^. However, the expression of p18 in PSCs is significantly lower than that in somatic fibroblasts (Fig. 1A,B). Mechanistically, the p18 protein can bind to Cdk4/6 and then prevent their interactions with cyclin D^15^,^23^,^24^, or block the phosphorylation of Cdk6 by CAK (CDK activation kinase)^25^. Thus, we propose that the relatively high level of p18 expression in somatic cells maintains the cell cycle through inactivation of Cdk4 and Cdk6. Ectopic expression of reprogramming factors induces down-regulation of p18 expression and therefore releases and activates Cdk4 and Cdk6, resulting in activation of genes promoting G1/S and G2/M transitions and DNA synthesis to facilitate somatic cell reprogramming (Fig. 6). Further experiments are required to dissect how reprogramming factors initiate inhibition of p18 expression during reprogramming.

In summary, our study highlights a novel pathway that restricts somatic cell reprogramming and provides a new target to optimize reprogramming efficiency.

## Materials and Methods

### Cell Culture

MEFs and 293 T cells were cultured in DMEM medium supplemented with 10% FBS (Gibco), 2 mM glutamine (Gibco), 1 mM sodium pyruvate (Gibco), and 0.1 mM β-mercaptoethanol (Sigma). ESCs and iPSCs were cultured in knockout DMEM medium supplemented with 15% FBS (Gibco), 2 mM glutamine (Gibco), 1 mM sodium pyruvate (Gibco), 0.1 mM β-mercaptoethanol (Sigma), 0.1 mM NEAA, P/S 100 U/mL(Gibco) and LIF. The culture medium was changed every other day.

All animal experiments were approved by the Ethics Committee in the Institute of Zoology, Chinese Academy of Sciences in accordance with the Guidelines for Care and Use of Laboratory Animals established by the Beijing Association for Laboratory Animal Science.

### Plasmids and antibody

The specific shRNA sequences targeting different genes are designed by a BLOCK-iT™ RNAi Designer (Thermo Fisher) and listed in Table S1. The oligos coding for different shRNAs were annealed and cloned into a pSicoR-GFP vector respectively. The cDNA sequences encoding p18 and p18^D68N^ were cloned into pMX. The reprogramming plasmids encoding mouse Oct4, Sox2, Klf4, and c-Myc were purchased from Addgene (pMXs-Oct3/4, 13366; pMXs-Sox2, 13367; pMXs-klf4, 13370; pMXs-c-Myc,13375). The anti-p18 antibody was from Santa Cruz (M-20; 1:250). The anti-actin antibody was purchased from Sigma (1/1000).

### Virus production

For reprogramming retrovirus preparation, the 10 μg of each retrovirus core vector and 10 μg of retrovirus package plasmid were co-transfected into 293 T cell per 10 cm dish using the Calcium Phosphate Transfection method. Media were changed 12 hour after transfection. Supernatants were collected 48 h after transfection, filtered by a 0.45-μm filter and then directly used for infection. The virus titers were monitored by co-transfection of pMX-GFP.

For different lentiviruses preparation, 10 μg of each lentiviral core vector and 7.5 μg of packaging vector psPAX2 and 5 μg packaging vector pMD2.G were co-transfected into 293 T cell per 10 cm dish using the Calcium Phosphate Transfection method. Supernatants were collected 48 h after transfection, filtered through a 0.45-μm filter, and used directly to infect MEFs. The virus titers were calculated by co-transfection of GFP.

### Generation of iPSCs

Around 5 × 10^4^ MEFs per well were infected with retroviral vector cocktails encoding the four reprogramming factors Oct4, Sox2, c-Myc and Klf4. 16 h after infection, the medium was replaced with fresh ESC medium and changed every day. Colonies with morphology similar to ESCs appeared approximately 12 days after reprogramming. Single iPSC colonies were picked and maintained in standard ESC culture medium. The reprogramming efficiency is around 0.01–0.5%.

### Western blot

Cells were washed twice with DPBS, lysed in RIPA buffer (Beyotime) supplement with protein inhibitors (Roche). Protein concentration is determined by a BCA protein assay kit (Beyotime). Protein sample were separated by SDS–PAGE electrophoresis and transferred to a PVDF membrane (Millipore). Then the membrane was incubated with non-fat milk diluted primary and secondary antibodies. The protein signals were detected by a Luminata Western HRP Substrates (Millipore).

### Quantitative real-time PCR

Total RNA was extracted from the indicated cells with an RNA Extraction Kit (GeneMark). cDNA was synthesized with oligo (dT) primers and Superscript Reverse Transcriptase (Life Technologies). Quantitative real-time PCR was performed with an ABI PRISM 7700 (Applied Biosystems), using a DNA Master SYBR Green I mix (Promega) according to the user manual. Housekeeping gene actin was used as a control. The relative gene expressions were calculated by the 2^−ΔΔCt^ method.

### Cell cycle analysis

Cells were washed three times with cold PBS, and then fixed in 70% ethanol at 4 °C for 2 h. After fixation, cells were washed with cold PBS twice and incubated with propidium iodide (PI) staining mixture (containing 200 mg/ml RNase A, 50 ug/ml PI) for 30 min at 37 °C in the dark. Cells were analyzed by a flow cytometry (BD Biosciences).

### Statistical analysis

All data in this study were analyzed with SPSS 13.0 and are presented as mean ± S.D. An independent sample t-test was used to determine the statistical differences between two groups. A one-way ANOVA was used to analyze multiple comparison procedures. Significant differences were considered as *P < 0.05, **P < 0.01 and ***P < 0.001.

## Additional Information

**How to cite this article**: Zhu, S. *et al*. p18 inhibits reprogramming through inactivation of Cdk4/6. *Sci. Rep.* **6**, 31085; doi: 10.1038/srep31085 (2016).

## Supplementary Material

Supplementary Information
