# Supplementary material for: p18 inhibits reprogramming through inactivation of Cdk4/6
Source: Sci Rep. 2016 Aug 3;6:31085. doi: 10.1038/srep31085 (PMC4971472; doi:10.1038/srep31085)
Supplement: Supplementary Information [file srep31085-s1.pdf]

p18 inhibits reprogramming through inactivation of Cdk4/6

Shaohua Zhu[1,2], Jiani Cao[2], Hongyan Sun[2,3], Kun Liu[2,3], Yaqiong Li[2,3], Tongbiao Zhao[1,2,3]*

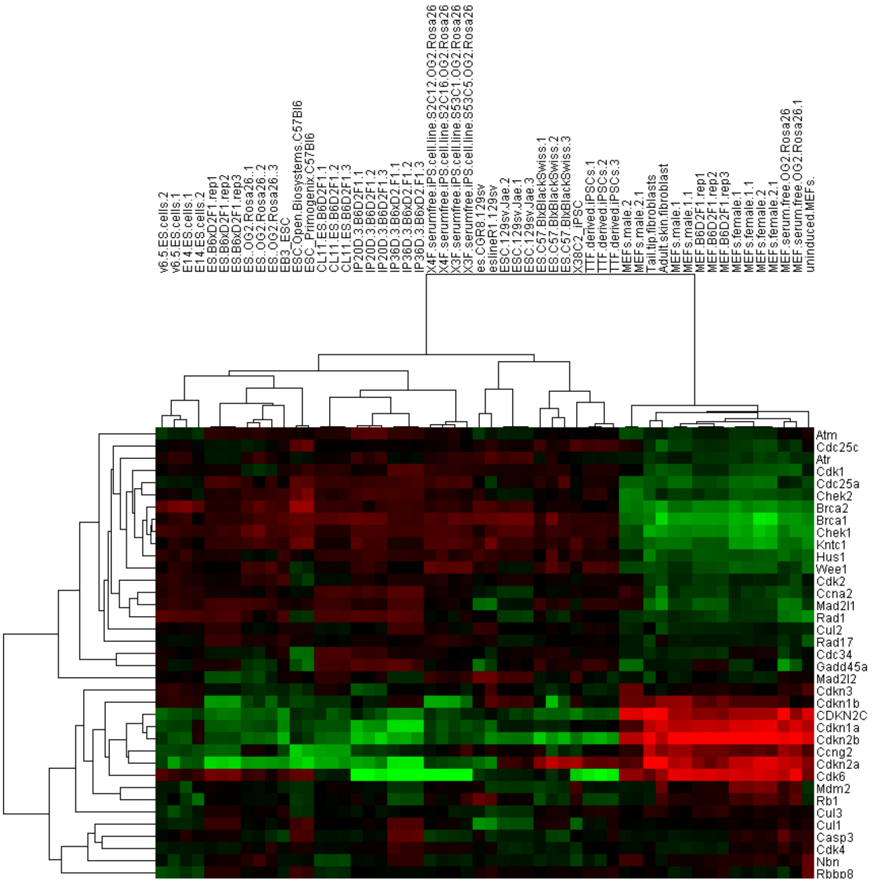

Supplementary Figure 1 Heat map of expression levels of the cell cycle related genes. 37 cell cycle relevant gene expressions were analyzed for their differential expression in mouse ESC, iPSC and somatic fibroblasts. The gene expression data were downloaded from NCBI. The GEO numbers of each cell line were listed in Supplementary table 2. R-Bioconductor was used for data quantile normalization and subsequent data processing. Genes, with p-value ≤0.05, false discovery rate (FDR) ≤ 0.05 and fold-change ≥ 2 (multiple hypothesis testing method, using the standard number of 1000 permutations), were considered as differentially expressed. Unsupervised hierarchical clustering of the expression profile was performed using Classter3.0 Software.

p18 inhibits reprogramming through inactivation of Cdk4/6

Shaohua Zhu[1,2], Jiani Cao[2], Hongyan Sun[2,3], Kun Liu[2,3], Yaqiong Li[2,3], Tongbiao Zhao[1,2,3]*

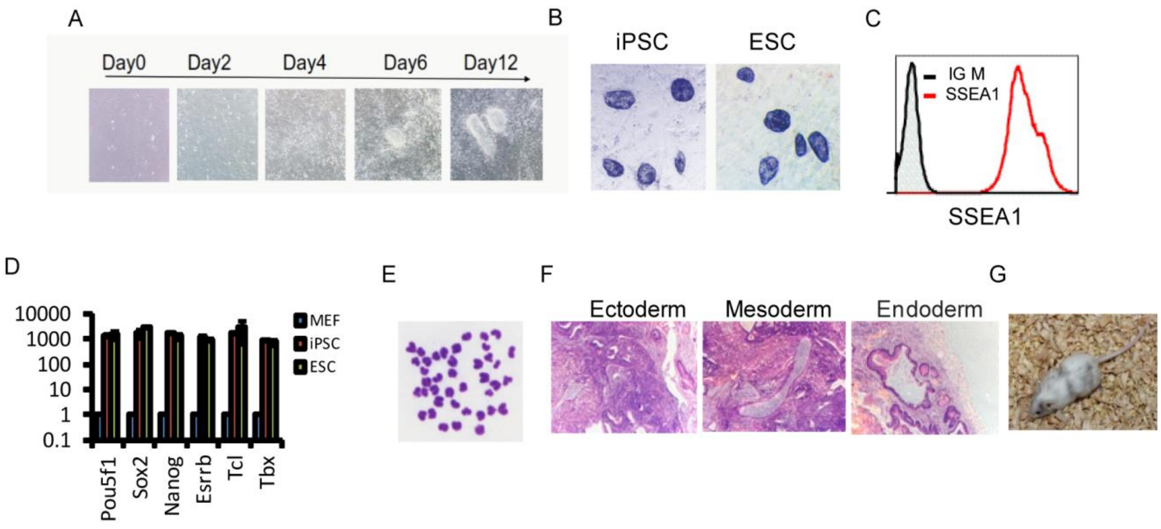

Supplementary Figure 2 The established iPSCs are pluripotent. A, Morphology of MEFs on days 0, 2, 4, 6 and 12 of reprogramming. **B,** Alkaline phosphatase staining of iPSCs and ESCs. **C,** The iPSCs express SSEA1. **D,** The iPSCs express pluripotency genes similar to ESCs. **E,** iPSCs have a normal karotype. **F,** iPSCs form teratomas containing all three embryonic germ layers in SCID mice. **G,** iPSCs contribute to chimeric mice.

p18 inhibits reprogramming through inactivation of Cdk4/6

Shaohua Zhu[1,2], Jiani Cao[2], Hongyan Sun[2,3], Kun Liu[2,3], Yaqiong Li[2,3], Tongbiao Zhao[1,2,3]*

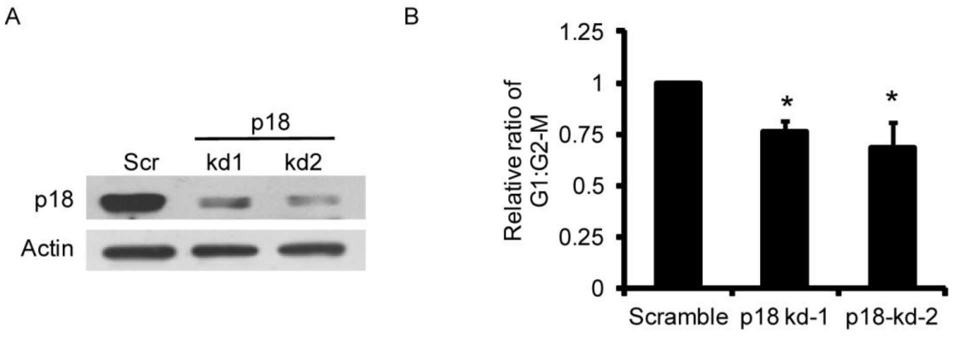

Supplementary Figure 3 Knock down of p18 enhance reprogramming. A, Western blot analysis of whole cell extracts from MEFs transfected with scramble or p18-specific shRNAs. Actin serves as a loading control. **B,** p18 knockdown significantly decreases the proportion of cells in G1 phase. Data are normalized to cells transfected with scramble shRNA and are shown as mean ± SD; n=3, * $P<0.05$.

p18 inhibits reprogramming through inactivation of Cdk4/6

Shaohua Zhu[1,2], Jiani Cao[2], Hongyan Sun[2,3], Kun Liu[2,3], Yaqiong Li[2,3], Tongbiao Zhao[1,2,3]*

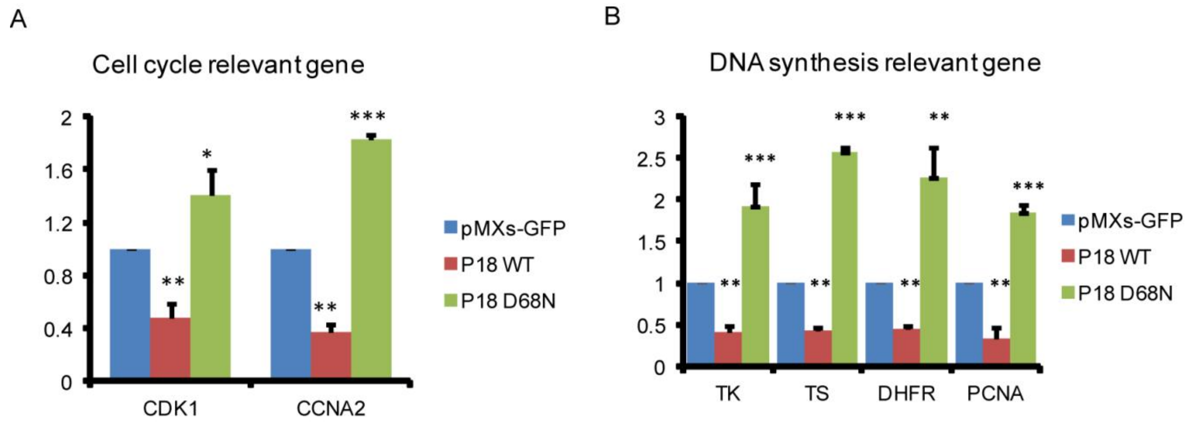

Supplementary Figure 4 Expression of Cdk4/6 target genes is inhibited by overexpression of wild-type p18 and enhanced by overexpression of D68N mutant p18. A, Expression of CDK1 and CCNA2 mRNAs in MEFs transfected with GFP, p18 or p18^{D68N}. The results are normalized to the GFP control and presented as the mean ± SD of three independent experiments; * P<0.05, ** P<0.01, *** P<0.001. B, Expression of TK, TS, DHFR and PCNA mRNAs in MEFs transfected with GFP, p18 or p18^{D68N}. The results are normalized to the GFP control and presented as the mean ± SD of three independent experiments; ** P<0.01, *** P<0.001.

p18 inhibits reprogramming through inactivation of Cdk4/6

Shaohua Zhu[1,2], Jiani Cao[2], Hongyan Sun[2,3], Kun Liu[2,3], Yaqiong Li[2,3], Tongbiao Zhao[1,2,3]*

Supplementary Table 1

**shRNA target sequence**		
	Sequences (5’ →3’)	
p18-sh1	Forward	GCTGCAGGTTATGAAACTTGG
	Reverse	CCAAGTTTCATAACCTGCAGC
P18-sh2	Forward	GCCGACCTTACTAGTATTTAC
	Reverse	GTAAATACTAGTAAGGTCGGC
Cdk4	Forward	GGCCTTTGAACATCCCAATGT
	Reverse	ACATTGGGATGTTCAAAGGCC
Cdk6	Forward	GGATATGATGTTTCAGCTTCT
	Reverse	AGAAGCTGAAACATCATATCC
**Primers for mouse genes relevant to cell cycle regulation and DNA synthesis**		
	Sequences (5’→3’)	
Actin	Forward	TGCTAGGAGCCAGAGCAGTA
	Reverse	AGTGTGACGTTGACATCCGT
p18	Forward	GGGGACCTAGAGCAACTTACT
	Reverse	AAATTGGGATTAGCACCTCTGAG
Ccna1	Forward	ACCGTGCTAGGGGTGTTGA
	Reverse	CGTTTGGCTGGTTCATTGACC
Cdk1	Forward	AGGTACTTACGGTGTGGTGTAT
	Reverse	CTCGCTTTCAAGTCTGATCTTCT
PCNA	Forward	TTGCACGTATATGCCGAGACC
	Reverse	GGTGAACAGGCTCATTCATCTCT
DHFR	Forward	CGCTCAGGAACGAGTTCAAGT
	Reverse	TGCCAATTCCGGTTGTTCAATAA
TS	Forward	GGAAGGGTGTTTTGGAGGAGT
	Reverse	GCTGTCCAGAAAATCTCGGGA
TK1	Forward	AGTGCCTGGTCATCAAGTATGC
	Reverse	GCTGCCACAATTACTGTCTTGC
Cdk6	Forward	TCTCACAGAGTAGTGCATCGT
	Reverse	CGAGGTAAGGGCCATCTGAAAA
Cdk4	Forward	AAGGTCACCCTAGTGTTTGAGC
	Reverse	CCGCTTAGAAACTGACGCATTAG

p18 inhibits reprogramming through inactivation of Cdk4/6

Shaohua Zhu[1,2], Jiani Cao[2], Hongyan Sun[2,3], Kun Liu[2,3],
Yaqiong Li2,3, Tongbiao Zhao1,2,3*

Supplementary Table 2

GPL1261 Title [Mouse430_2] Affymetrix Mouse Genome 430 2.0 Array

sample	GSE	GSM
ES.B6xD2F1.rep1	GSE21515	GSM537476
ES.B6xD2F1.rep2	GSE21515	GSM537477
ES.B6xD2F1.rep3	GSE21515	GSM537478
v6.5.ES.cells.1	GSE14012	GSM344757
v6.5.ES.cells.2	GSE14012	GSM344758
E14.ES.cells.1	GSE14012	GSM344759
E14.ES.cells.2	GSE14012	GSM344760
ES..OG2.Rosa26..1	GSE10806	GSM272753
ES..OG2.Rosa26..2	GSE10806	GSM272836
ES..OG2.Rosa26..3	GSE10806	GSM272837
es.CGR8.129sv	GSE15267	gsm381701
eslineR1.129sv	GSE15267	gsm381704
ESC.Open.Biosystems.C57Bl6	GSE44923	GSM1093945
ESC_Primogenix.C57Bl6	GSE44923	GSM1093946
ES.C57.BlxBlackSwiss.1	GSE32598	GSM808020
ES.C57.BlxBlackSwiss.2	GSE32598	GSM808021
ES.C57.BlxBlackSwiss.3	GSE32598	GSM808022
EB3_ESC	GSE31725	GSM784988
CL11.ES.B6D2F1.1	GSE16925	GSM424475
CL11.ES.B6D2F1.2	GSE16925	GSM424476
CL11.ES.B6D2F1.3	GSE16925	GSM424477
ESC.129sv.Jae.1	GSE24705	GSM638103
ESC.129sv.Jae.2	GSE24705	GSM638104
ESC.129sv.Jae.3	GSE24705	GSM638105
MEFs.male.1	GSE14012	gsm344765
MEFs.male.2	GSE14012	gsm344766
MEFs.female.1	GSE14012	gsm344767
MEFs.female.2	GSE14012	gsm344768
Tail.tip.fibroblasts	GSE32598	GSM808023
Adult.skin.fibroblast	GSE31725	GSM784982
MEF.B6D2F1.rep1	GSE16925	GSM424478
MEF.B6D2F1.rep2	GSE16925	GSM424479
MEF.B6D2F1.rep3	GSE16925	GSM424480
MEF.serum.free.OG2.Rosa26	GSE15267	GSM381302
MEF.serum.free.OG2.Rosa26.1	GSE15267	GSM381303
uninduced.MEFs.	GSE14012	gsm344756
MEFs.male.1.1	GSE14012	gsm344765
MEFs.male.2.1	GSE14012	gsm344766
MEFs.female.1.1	GSE14012	gsm344767
MEFs.female.2.1	GSE14012	gsm344768
X4F.serumfree.iPS.cell.line.S2C12.OG2.Rosa26	GSE15267	GSM381305
X4F.serumfree.iPS.cell.line.S2C16.OG2.Rosa26	GSE15267	GSM381306
X3F.serumfree.iPS.cell.line.S53C1.OG2.Rosa26	GSE15267	GSM381307
X3F.serumfree.iPS.cell.line.S53C5.OG2.Rosa26	GSE15267	GSM381308

IP20D.3.B6D2F1.1	GSE16925	GSM424487
IP20D.3.B6D2F1.2	GSE16925	GSM424488
IP20D.3.B6D2F1.3	GSE16925	GSM424489
X38C2_iPSC	GSE31725	GSM784983
TTF.derived.iPSCs.1	GSE32598	GSM808017
TTF.derived.iPSCs.2	GSE32598	GSM808018
TTF.derived.iPSCs.3	GSE32598	GSM808019
IP36D.3.B6xD2.F1.1	GSE21515	GSM537488
IP36D.3.B6xD2.F1.2	GSE21515	GSM537489
IP36D.3.B6xD2.F1.3	GSE21515	GSM537490
